# Optimizing intensive care capacity using individual length-of-stay prediction models

**DOI:** 10.1186/cc5730

**Published:** 2007-03-27

**Authors:** Mark Van Houdenhoven, Duy-Tien Nguyen, Marinus J Eijkemans, Ewout W Steyerberg, Hugo W Tilanus, Diederik Gommers, Gerhard Wullink, Jan Bakker, Geert Kazemier

**Affiliations:** 1 Department of Operating Rooms, Erasmus University Medical Center, P.O. Box 2040, 3000 CA Rotterdam, The Netherlands; 2 Department of Public Health, Erasmus University Medical Center, P.O. Box 2040, 3000 CA Rotterdam, The Netherlands; 3 Department of Surgery, Erasmus University Medical Center, P.O. Box 2040, 3000 CA Rotterdam, The Netherlands; 4 Department of Anesthesiology, Erasmus University Medical Center, P.O. Box 2040, 3000 CA Rotterdam, The Netherlands; 5 Department of Intensive Care, Erasmus University Medical Center, P.O. Box 2040, 3000 CA Rotterdam, The Netherlands

## Abstract

**Introduction:**

Effective planning of elective surgical procedures requiring postoperative intensive care is important in preventing cancellations and empty intensive care unit (ICU) beds. To improve planning, we constructed, validated and tested three models designed to predict length of stay (LOS) in the ICU in individual patients.

**Methods:**

Retrospective data were collected from 518 consecutive patients who underwent oesophagectomy with reconstruction for carcinoma between January 1997 and April 2005. Three multivariable linear regression models for LOS, namely preoperative, postoperative and intra-ICU, were constructed using these data. Internal validation was assessed using bootstrap sampling in order to obtain validated estimates of the explained variance (r^2^). To determine the potential gain of the best performing model in day-to-day clinical practice, prospective data from a second cohort of 65 consecutive patients undergoing oesophagectomy between May 2005 and April 2006 were used in the model, and the predictive performance of the model was compared with prediction based on mean LOS.

**Results:**

The intra-ICU model had an r^2 ^of 45% after internal validation. Important prognostic variables for LOS included greater patient age, comorbidity, type of surgical approach, intraoperative respiratory minute volume and complications occurring within 72 hours in the ICU. The potential gain of the best model in day-to-day clinical practice was determined relative to mean LOS. Use of the model reduced the deficit number (underestimation) of ICU days by 65 and increased the excess number (overestimation) of ICU days by 23 for the cohort of 65 patients. A conservative analysis conducted in the second, prospective cohort of patients revealed that 7% more oesophagectomies could have been accommodated, and 15% of cancelled procedures could have been prevented.

**Conclusion:**

Patient characteristics can be used to create models that will help in predicting LOS in the ICU. This will result in more efficient use of ICU beds and fewer cancellations.

## Introduction

Intensive care units (ICUs) consume a considerable portion of hospital budgets. Moreover, costs are predicted to rise with the emergence of new treatment methods. Problems with ICU capacity are nevertheless common, and studies conducted in ICUs have documented high rates of refusal to admit because of lack of empty beds [1,2]. In addition, the need to serve the 'greying' population is likely to increase demand for ICU beds further, exacerbating the current strain on ICU capacity. Consequently, hospitals will face an increase in numbers of cancelled surgical procedures that necessitate postoperative intensive care, and higher rates of refusal to admit other critically ill patients [2,3]. The only way to remedy these problems is apparently to improve the efficiency with which the available ICU and operating room capacity is used, in other words to optimize patient planning.

Patient planning depends importantly on reliable and adequate management information. Key elements in the ICU setting are the patient's expected length of stay (LOS) in the ICU at admission and possible changes in expected LOS resulting from later treatment. Starting from the admission date and expected LOS, the planner will be able to pinpoint the anticipated date at which an ICU bed will once again become available. This information, along with subsequent changes in a patient's expected LOS, is needed to schedule the next operating room patient who requires postoperative intensive care or to reserve emergency patient capacity on the ICU. In addition, information on expected LOS preoperatively facilitates scheduling of individual surgical procedures on specific dates. This information can be used to predict ICU admission dates and LOSs. Information that emerges during the surgical procedure and the postoperative stay in the ICU can influence the LOS predicted by the preoperative model. This so-called online patient planning can help to improve OR and ICU programmes.

Clinicians generally assume that LOS of individual patients is unpredictable. Intensivists are expected to be able predict LOS roughly, but the accuracy of this prediction depends largely on the intensivist's experience. We speculate that if comprehensive evaluations of the association between preoperative, intraoperative and postoperative prognostic variables on the one hand, and LOS on the other are translated into a mathematical model, then this model might be able to predict LOS with greater accuracy.

The main goal of this study was to develop a model that will provide planners with a tool to predict the LOS of individual patients in the ICU. Data on a cohort of consecutive patients undergoing an elective oesophagectomy were used to create and validate such models. Predictive power was assessed to determine the best performing model. In a second cohort of patients, the LOSs of individual patients were predicted prospectively to determine the potential gain of this best model on a day-to-day basis.

## Methods

### Data

Data from 518 consecutive patients who underwent elective oesophagectomy with reconstruction for carcinoma at the Erasmus University Medical Centre, Rotterdam, The Netherlands, between January 1997 and April 2005, were retrieved from the hospital information system. These data were combined with detailed data from a prospective database held at the Department of Surgery. The Erasmus University Medical Centre includes a total of 1,212 beds on several locations. It is a trauma centre for a catchment area that includes 5.2 million people. The main site includes 32 ICU beds and 19 operating rooms.

The outcome variable of the present study was LOS, defined as the time in days between admission and discharge from the ICU. Admission to and discharge from the ICU were based on the national protocol [4]. For patients discharged to the ward and readmitted to the ICU within 48 hours, the intervening stay on the ward was included in the LOS. Definitions of these variables and supporting references [5-7] are given in Table 1; reports that provide evidence supporting the use of these variables in the model are also referenced [8-12].

**Table 1:** Characteristics for both cohorts of patients who underwent oesophagectomy with reconstruction for cancer

	Construction sample (*n *= 518)	Application sample (*n *= 65)	Reference
Patient characteristics			

Age (years)	63 (55–70)	60 (56–68)	[8-12]
Male sex	407 (79)	48 (74)	[9-12]
BMI (kg/m^2^)	25 (22–28)	26 (23–29)	[8]
ASA 1, 2	89 (17)	28 (43)	[5]
Hypertension	192 (37)	35 (54)	[6]
Previous stomach operation	132 (25)	19 (29)	[6]
Preoperative serum haemoglobin (mmol Fe/l)	8.4 (7.6–9.2)	8.7 (7.4–9.4)	[11,12]
Preoperative serum creatinin (μmol/l)	78 (68–89)	78 (68–90)	[11,12]
Preoperative FEV_1 _(l)	2.9 (2.4–3.5)	3.2 (2.4–3.7)	
Preoperative chemotherapy	170 (33)	17 (26)	[8,10]
Preoperative radiotherapy	55 (11)	8 (12)	[8,10]
Aetiology			

Gastroesophageal reflux disease	63 (12)	9 (14)	[6]
Barrett's esophagus	43 (8)	9 (14)	[6]
Other	66 (13)	13 (20)	[6]
Comorbidities			

Cardiac	134 (26)	24 (37)	[6]
Respiratory	91 (17)	7 (11)	[6]
Vascular	65 (13)	6 (9)	[6]
Neurological	33 (6)	7 (11)	[6]
Diabetes mellitus	51 (10)	7 (11)	[6]
Other carcinoma	53 (10)	4 (6)	[6]
Other	40 (8)	2 (3)	[6]
Tumour characteristic			

Adenocarcinoma	340 (66)	51 (79)	
pTNM stage 0	27 (5)	7(11)	[7]
I	64 (12)	5 (8)	[7]
IIa	120 (23)	13 (20)	[7]
IIb	46 (9)	5 (8)	[7]
III	193 (37)	21 (32)	[7]
IV	68 (13)	14 (22)	[7]
Radicality (R0)	400 (77)	54 (83)	
Session variables			

Expected duration of the procedure (min)	240 (180–270)	266 (262–314)	
Duration of the procedure (min)	301 (254–359)	333 (290–368)	[11]
Total age of the two head surgeons (years)	83 (72–88)	84 (74 – 94)	
Transthoracic approach	114 (22)	14 (22)	[8, 10–12]
Reconstruction using colon	24 (5)	3 (5)	[10–12]
Oesophagus and cardia resection	506 (98)	65 (100)	[6]
Splenectomy during surgical procedure	15 (3)	2 (3)	
Absolute crystalloid administration (l)	6.0 (4.5–7.0)	4.0 (2.3–5.5)	[8]
Absolute colloid administration (l)	1.5 (1.5–2.0)	1.5 (1.5–2.0)	[8]
Erythrocyte concentrate transfusion	276 (53)	22 (33)	[8,12]
Fresh frozen plasma transfusion	36 (7)	6 (10)	[8,12]
Absolute blood loss (l)	1.5 (1.0–2.2)	1.1 (0.7–1.5)	[8,12]
Absolute urine production (l)	0.7 (0.4–1.3)	0.4 (0.3–0.7)	
Epidural analgesia during procedure	467 (90)	57 (88)	
Vasopressor administration	214 (41)	63 (97)	
Duration of vasopressor therapy (hours)	0 (0–1.5)	270 (206–337)	
Minute volume (l)	7.8 (7.2–8.8)	7.8 (7.0–8.4)	
Positive end-expiratory pressure (cmH_2_O)	5 (4–7)	6 (5–7)	
Serum oxygen saturation (%)	98 (96–100)	100 (98–100)	
End temperature (°C)	35.8 (35.2–36.4)	36.3 (36.0–36.9)	
Lactate (mmol/l)	1.7 (1.2–2.2)	1.4 (1.0–2.0)	
Postoperative variables			

Duration of mechanical ventilation	0.63 (0.13–5.58)	0.54 (0.12–4.12)	
Surgical complications			
Postoperative bleeding	19 (4)	1 (2)	[6]
Chylothorax	20 (4)	3 (5)	[6]
Leakage of anastomosis	38 (7)	9 (13)	[6]
Necrosis of anastomosis	18 (4)	2 (3)	[6]
Other	42 (8)	11 (17)	[6]
Nonsurgical complications			
Pulmonary: pneumonia, atelectasis, or ARDS	198 (38)	24 (37)	[6]
Infection: urinary tract, sepsis	31 (6)	1 (2)	[6]
Thrombosis, embolism	20 (4)	3 (5)	[6]
Other	135 (26)	26 (40)	[6]
Length of stay in the ICU (days)	4.0 (2.0–7.9)	4.2 (2.9–7.9)	

Values are expressed as number (%) or, for continuous variables, as median (25th to 75th percentile). ARDS, acute respiratory distress syndrome; ASA, American Society of Anaesthesiology Physical Status Score; BMI, body mass index; FEV1, forced expiratory volume in 1 s; ICU, intensive care unit.

### Model construction

Only those variables that were present in more than 1% of the patients were included, in order to avoid unstable estimates. On clinical grounds, two expert surgeons (HWT and GK) and two expert anaesthetists (DG and JB) formed a preselection of factors from the potentially prognostic variables in order to prevent overfitting [13-15]. Only these selected variables were used to build the three models. In those patients with missing values, data were completed using multiple imputation methods. This was done under the assumption that the distribution of the missing date and the complete data were the same [16].

The imputed model included both the independent potentially prognostic variables and the outcome variable LOS. Given the inherently skewed distribution of LOS, a natural log transformation was used [17].

Univariate linear regression analysis was used to test which of the variables contributed to LOS with *P *≤ 0.20. The mean and standard deviation are reported for those variables that are normally distributed. The median and interquartile ranges are given for non-normal distributions.

Significant variables in the univariate analyses were entered as potentially prognostic variables into a backward, stepwise selection procedure to construct a multivariable linear model that provides a natural logarithm transformed prediction of LOS (ln [LOS]). Because LOS can be predicted based on expanding sets of available information at three stages, three multivariable linear models were constructed. First, preoperative data were used to build a preoperative prediction model. Then, intraoperative data were incorporated to construct a postoperative model. To construct an intra-ICU model, which was used after three days on the ICU, all selected data were used. This final model was constructed to improve accuracy based on new information from the last three ICU days. The criterion for retention of variables in the model was *P *< 0.20, which ensured high power for inclusion of variables with somewhat weaker predictive effects [14]. Interactions between variables and nonlinear relationships were explored. A smearing factor to correct the 'back transformation' bias was needed to obtain the estimated LOS, because a natural logarithmic transformation on LOS was used [18]. Goodness-of-fit was assessed graphically by plotting observed LOS against predicted LOS in a calibration plot. The predictive power of the model was expressed as the percentage explained variation (multiple r^2^) on the logarithmic scale.

Internal validity was assessed with bootstrap sampling to obtain estimates of the optimism of the multiple r^2 ^[14,19,20]. This optimism indicates the expected decrease in model performance when it is applied in future patients [21]. Bootstrap samples were drawn with replacement and with the same size as the original sample. Regression models were constructed in each bootstrap sample and tested on the original sample. This was repeated 200 times to obtain stable estimates of the optimism of the model [21].

Analyses were performed using SPSS version 11 (SPSS Inc, Chicago, IL, USA) and S-Plus version 6 (Insightful Inc, Seattle, WA, USA).

### Model application

After internal validation of the models, the gain in terms of usage of ICU capacity with the model exhibiting the highest r^2 ^was assessed in routine clinical practice. Prospective data were collected for consecutive patients who underwent elective oesophagectomy with reconstruction for carcinoma. The data were collected during the period from May 2005 to April 2006, which were the 12 months after construction of the model (Table 1). The prediction model was assessed by comparing the total overestimation and underestimation of the required ICU days if the mean LOS was used (the old situation) with the total overestimation and underestimation of the required ICU days if the prediction model was used (the new situation). The overestimation and underestimation in the old situation were calculated by subtracting the observed LOS from the mean LOS. The overestimation and underestimation in the new situation were calculated by subtracting the observed LOS from the predicted LOS. The mean LOS was used rather than the median LOS, because use of the median will favour the prediction model because the LOS is skewed. Therefore, use of the mean LOS will result in a more conservative gain in comparison with the median LOS.

Both the old and new situations have three possible outcomes: negative, indicating that the ICU bed was reserved for too long and that the number of ICU days was overestimated; zero, indicating perfect prediction; or positive, indicating that the ICU bed was reserved for an insufficient period and that the number of ICU days was underestimated. The total overestimation and the underestimation were calculated for both the mean LOS approach and the LOS prediction model for both the old and the new situations.

## Results

### Retrospective population

**Figure 1:**
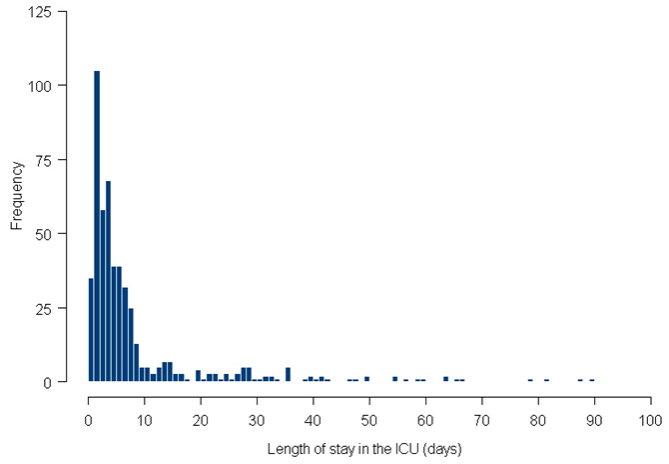
Distribution of length of stay in the ICU. ICU, intensive care unit.

The mean LOS was 8.76 days and the median LOS was 4.0 days (interquartile range 2.0 to 7.9 days). Overall, 6.8% of the patients were discharged from the ICU within 1 day after their surgical procedure, 37% within 3 days, 56% within 5 days and 69% within 7 days (Figure 1). Thirty-eight patients (7.3%) were readmitted to the ICU after a stay shorter than 48 hours on the ward. Table 1 lists the retrieved data for variables that were thought to be potentially prognostic, broken down into patient characteristics, tumour and session characteristics, and postoperative complications within the first 72 postoperative hours. ICU mortality was 2.5% and total in-hospital mortality was 4.1%.

### Univariate analysis

The following preoperative variables (Table 1) were associated with longer LOS: older age (*P *< 0.001), American Society of Anesthesiology's Physical Status 3 or 4 (*P *= 0.001), presence of five out of seven comorbidities (*P *< 0.001 to 0.14), squamous cell carcinoma (*P *= 0.003), transthoracic approach instead of transhiatal (*P *< 0.001), reconstruction using colon instead of stomach (*P *= 0.02), previous chemotherapy (*P *= 0.003) and lower forced expiratory volume in 1 s during preoperative screening (*P *< 0.001). Intraoperative variables associated with longer LOS were higher absolute amount of colloids administered (*P *= 0.01), greater absolute blood loss (*P *= 0.04), longer duration of vasopressor administration (*P *= 0.03), higher respiratory minute volume (*P *= 0.005) and lower arterial oxygen saturation (*P *< 0.001). Patients with any complication occurring within 72 hours after surgery also had significantly longer LOS (*P *< 0.001).

### Preoperative, postoperative and intra-ICU multivariable models

The multiple r^2 ^for the preoperative model was 21% and the optimism was 6%; hence, the r^2 ^after validation was 15%. The preoperative model had a 95% confidence interval (CI) with relative bounds between 0.5 and 2.5. This implies that LOS may be from 50% shorter to 254% longer than the mean LOS. Patient age (*P *= 0.001), presence of gastroesophageal reflux disease (*P *< 0.001), neurological comorbidity (*P *< 0.001) and a transthoracic instead of transhiatal approach (*P *< 0.001) were the variables that contributed most to the increase in LOS for the preoperative model.

For the postoperative model, the multiple r^2 ^was 25% and the optimism was 9%; the r^2 ^after validation was 17%. The 95% CI with relative bounds was comparable to that of the preoperative model. Apart from the variables included in the preoperative model, higher absolute amount of colloids administered (*P *= 0.03) and a maximum respiratory minute volume during the surgical procedure (*P *< 0.001) were the variables found to contribute to LOS in the postoperative model.

The multiple r^2 ^of the intra-ICU model was 56% and the optimism was 11%, resulting in an r^2 ^of 45% after validation. The intra-ICU model had a 95% CI with relative bounds between 0.3 and 3.4, implying that LOS may be from 70% shorter to 340% longer than the mean LOS. Complications occurring within 72 hours in the ICU (five complications had *P *< 0.001 and two complications had *P *< 0.06) were the variables found to contribute to LOS in the intra-ICU model. Results are shown in Table 2 and formulas to calculate the LOS of the preoperative, postoperative, and intra-ICU models can be found in Additional files 1, 2, and 3, respectively.

**Table 2:** Multivariable preoperative, postoperative and intra-ICU linear LOS analyses

	Preoperative model	Postoperative model	Intra-ICU model
(Constant)	1.26	0.44	1.82
Expected session time (min)	1.10 (1.01–1.21)		
Patient age (per decade)	1.16 (1.06–1.28)	1.20 (1.09–1.31)	1.09 (1.02–1.17)
FEV_1 _(l)	0.91 (0.81–1.03)	0.85 (0.75–0.96)	
Gastroesophageal reflux disease (yes/no)	1.46 (1.14–1.89)	1.53 (1.19–1.96)	
Vascular comorbidity (yes/no)	1.29 (1.01–1.66)	1.32 (1.03–1.69)	
Neurological comorbidity (yes/no)	1.74 (1.24–2.43)	1.82 (1.31–2.53)	
Previous chemotherapy (yes/no)	0.81 (0.68–0.97)		
Previous radiotherapy (yes/no)			0.78 (0.63–0.97)
Transthoracic approach (yes/no)	2.13 (1.74–2.62)	1.79 (1.44–2.24)	1.21 (1.05–1.40)
Reconstruction using colon (yes/no)	1.56 (1.05–2.30)	1.52 (1.03–2.23)	
Observed session time (min)		1.07 (0.98–1.16)	
Volume administration of colloids (liter)		1.14 (1.01–1.29)	
Absolute intraoperative blood loss (l)		0.94 (0.87–1.02)	
Absolute intraoperative urine production (l)		1.12 (0.99–1.25)	
Epidural analgesia administration (yes/no)		0.83 (0.69–1.01)	
Respiratory minute volume (l)		1.09 (1.04–1.15)	1.05 (1.01–1.09)
Positive end-expiratory pressure (cmH_2_O)		1.03 (0.99–1.07)	
Chylothorax surgical complication (yes/no)			1.31 (0.96–1.79)
Anastomosis leakage complication (yes/no)			1.83 (1.47–2.28)
Other complication (yes/no)			1.71 (1.38–2.10)
Pulmonary nonsurgical complication (yes/no)			1.97 (1.72–2.26)
Myocardial infarction (yes/no)			1.54 (0.93–2.56)
Infection (yes/no)			1.61 (1.25–2.07)
Other nonsurgical complication (yes/no)			1.41 (1.22–1.62)
Multiple r^2^	21%	25%	56%
Optimism	6%	9%	11%
Optimism corrected r^2^	15%	17%	45%

Unless stated otherwise, values are expressed as coefficient (95% confidence interval). FEV1, forced expiratory volume in 1 s; ICU, intensive care unit; LOS, length of stay.

**Figure 2:**
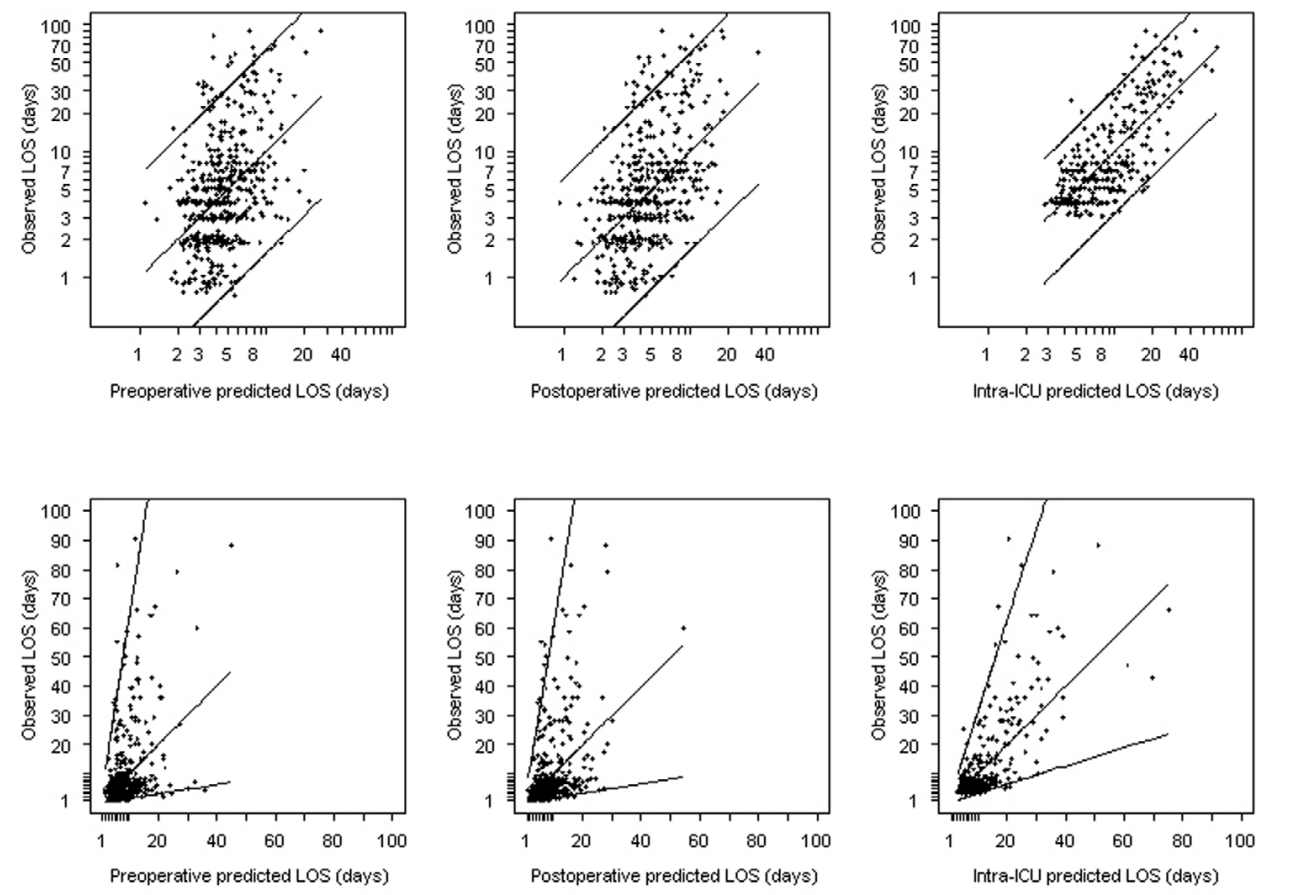
Calibration plots of the observed LOS against predicted LOS. These plots were constructed using the multivariable preoperative, postoperative and intra-ICU linear LOS models, on logarithmic scales (upper panel) and on untransformed scale with smearing factor (lower panels). ICU, intensive care unit; LOS, length of stay.

The goodness-of-fit of the three models is shown in Figure 2, which reveals considerable variation. Preoperative and postoperative LOS predictions exhibit variation and are not symmetrically distributed around the regression line. The LOS predictions of the intra-ICU model vary less, however, and are symmetrical. In addition, the prediction bounds of the intra-ICU model are much smaller than those of the preoperative and postoperative models.

### Model application

Because the intra-ICU model has the highest r^2^, this model was assessed for the second, prospective cohort of patients. This model included only patients who stayed in the ICU for at least three days. Of the 65 patients, 46 had a LOS longer than three days. The mean LOS for patients staying longer than three days in the ICU was 14.6 days, and so the 'remaining mean LOS' of 11.6 days (after subtraction of the first 3 ICU days) was used for comparison with the intra-ICU model. In the old situation (remaining mean LOS) and the new situation (prediction model), 10 out of 46 patients had an observed LOS longer than predicted (underestimation of ICU days), and the remaining 36 had an observed LOS shorter than predicted (overestimation of ICU days). In the old situation, these 10 patients together accounted for an underestimation of 220 ICU days; in the new situation they accounted for an underestimation of 155 ICU days. The other 36 patients together occupied the ICU for 213 days longer than predicted in the old situation, but in the new situation they occupied the ICU for 236 days longer than predicted (Table 3).

**Table 3:** Application of the model: underestimation and overestimation of old and new situation

	Situation	
	
	Old	New
Patients with underestimation (*n*)	10	10
Total underestimated days	220	155
Patients with overestimation (*n*)	36	36
Total overestimated days	213	236
Difference in underestimated days	-	65
Difference in overestimated days	-	23

In the old situation, estimation of LOS was based on the remeaning LOS after three days. The new situation used the prediction model. LOS, length of stay.

All in all, the total underestimation of ICU days decreased by 65 in favour of the prediction model; this is equal to 11% of the total ICU capacity of the study group. The total overestimation ICU days increased by 23 with the prediction model (in favour of prediction based on mean LOS). LOS was underestimated by the prediction model in 10 patients; this underestimation was less than in the old situation, however. Ultimately, 10 patient cancellations were prevented, which is equivalent to 15% of included patients.

## Discussion

We showed that a predictive model incorporating characteristics of individual patients who underwent oesophagectomy for cancer enhanced the accuracy of estimated LOS. Key prognostic variables included patient's age, presence of gastroesophageal reflux disease, respiratory minute volume, transthoracic rather than transhiatal approach, and complications within the first 72 postoperative hours. We assessed three models and found that the intra-ICU model, which uses data from the first 72 hours in the ICU, had the best predictive performance. We found that use of this model in our clinical setting would have resulted in a gain of 65 ICU days over a 12-month period. This is equivalent to 11% of the ICU capacity for this patient group. Moreover, 15% of cancellations of future surgical procedures could have been prevented.

Three types of related studies have been reported in the literature. First, we found reports of LOS prediction models that suggest specific therapeutic interventions in patient groups that may influence LOS [22,23]. Second, prediction models to determine risks for prolonged LOS have been developed [24-31]. In these studies the investigators used preoperative, intraoperative and postoperative variables to fit a logistic model, with risk for prolonged LOS as the main outcome. This outcome measure is claimed to improve planning and therefore cost-effectiveness of hospitals. However, the results from these studies do not permit scheduling of individual patients on the ICU. They only calculate the risk for prolonged LOS given a certain cutoff point. The third type of study also uses individual patient characteristics to predict LOS, as in our study. However, they apply less sophisticated mathematical techniques (multiple linear regression), whereas the present study used logistic regression [32]. These models can only be used for medically homogeneous patient groups with a shorter and less variable LOS. In summary, the models proposed in these earlier studies are unsuitable for scheduling of individual patients in the ICU. In contrast, the prediction model proposed here does permit individual patient scheduling in the ICU on a day-to-day basis.

A typical example illustrates the value of the prediction model. A 79-year-old oesophagectomy patient without previous radiotherapy was operated on via a transhiatal approach; the measured maximum respiratory minute volume was 9.6 l during the surgical procedure, and various complications occurred within the first 72 hours in the ICU. The predicted LOS for that patient using our model is 84 days. This well exceeds the mean LOS of 8.76 days that was calculated using data in the hospital information system.

Prediction models, such as that proposed here, can improve quality of care and cost-effectiveness in an ICU, as was demonstrated in the prospective second cohort of patients we analyzed. Data required for the development and application of prediction models are typically available in every hospital. Therefore, prediction models can be used in almost any clinical setting, but they must be developed for specific groups of individual patients if the full benefit in terms of capacity gain is to be realized. The ICU typically occupies an important position in patient flow, and discharge of a patient typically allows new patients to enter the ICU. More accurate prediction of ICU discharge date therefore results in a more reliable and predictable care process, not just in the ICU but throughout the patient care pathway, including the operating room and the ward.

There are some limitations to our study. It was conducted among just one group at a single centre, which may limit the generalizability of our results to other centres. In addition, classification of variables will not be the same in all centres. The development of models like those proposed here requires effort. Also, some variables may change over time, and so the model should be updated periodically to maintain accuracy of prediction. Moreover, data such as pathological stage and how radical the surgical procedure is are typically only available during the second week postoperatively at our hospital, and so this information cannot be used as variables in a model during the first week. The extent of lymph node dissection was standardized in the surgical approach, and so an extra variable was not needed for this type of operation [33]. In the present study, the mean LOS at the ICU after oesophagectomy was long. The majority of patients had a LOS of more than 3 days. Estimates of ICU LOS in the literature vary, but the ICU LOS at our institution appears to be reasonable in comparison with those reports [8,34,35]. Clearly, ICU LOS prediction models are of greater value to hospitals with patient groups that have longer mean LOS. In our study, the particular patient group chosen was selected for that reason, so that we could experiment with the creation of such a model.

A strength of our study is that there is no selection bias; all patients were admitted to the ICU postoperatively according to protocol. Although our application sample differs statistically from the construction sample for some variables, the model appeared robust enough to make accurate predictions. Furthermore, a multiple imputation method was used to impute missing values, and so all patients were indeed included in the analysis.

## Conclusion

We constructed, validated and tested three models, with incrementally enhanced precision, to predict LOS for individual patients in the ICU. The intra-ICU model proved able to predict LOS most accurately. For the highly variable LOS of oesophagectomy patients, this model appears to counter the commonly held view that LOS is unpredictable. Moreover, comparing the predictions of the model with historically determined mean LOS yielded significant improvement in terms of ICU capacity.

## Key messages

• It is possible to construct a model to predict LOS in the ICU for an individual patient undergoing oesophagectomy with reconstruction for carcinoma, which has accuracy superior to that of prediction based on the mean LOS.

• These models can improve scheduling of patients in the ICU, yielding more efficient use of ICU beds and better quality of care as a result of fewer cancellations.

• Other models should be developed for other elective surgical procedures that require postoperative intensive care in order to improve efficiency and quality of care in the ICU.

## Abbreviations

CI = confidence interval; ICU = intensive care unit; LOS = length of stay.

## Competing interests

The authors declare that they have no competing interests.

## Authors' contributions

MH, DTN, MJE, GW and GK made contributions to the concept, design and acquisition of data. DTN, MJE and EWS were responsible for the analysis and interpretation of data. HWT, DG, JB and GK were involved with clinical aspects of the study. All authors read and approved the final manuscript.

## Supplementary Material

Additional file 1 A Word document showing calculation of the ICU LOS using the preoperative prediction model. Click here for file

Additional file 2 A Word document showing calculation of the ICU LOS using the postoperative prediction model. Click here for file

Additional file 3 A Word document showing calculation of the ICU LOS using the intra-ICU prediction model. Click here for file
