# Supplementary material for: Optimizing intensive care capacity using individual length-of-stay prediction models
Source: Crit Care. 2007 Mar 27;11(2):R42. doi: 10.1186/cc5730 (PMC2206463; doi:10.1186/cc5730)
Supplement: Additional file 1 — A Word document showing calculation of the ICU LOS using the preoperative prediction model. [file cc5730-S1.doc]

**Appendix A**

Length Of Stay in the Intensive Care Unit (ICU) (LOS) with the preoperative prediction model can be calculated as:

LOS_preoperative_ (days) = 1.26 * 1.10^exp * 1.16^age/10 * 0.91^fev * 1.46^gerd * 1.29^vasc * 1.74^neu * 0.81^che * 2.13^tte * 1.63,

where exp is the estimate of the surgeon for the session time per minute; age is patient's age per year; fev is the forced expiratory volume 1 (FEV1, see Table 1) in liters; gerd is 1, if patient has gastroesophageal reflux disease, 0 if not; vasc is 1, if patient has a vascular comorbidity, 0 if not; neu is 1, if patient has a neurological comorbidity, 0 if not; che is 1, if patient had chemotherapy, 0 if not; tte is 1, if transthoracic approach, 0 if transhiatal approach; and 1.63 is the smearing factor.
