# Supplementary material for: Optimizing intensive care capacity using individual length-of-stay prediction models
Source: Crit Care. 2007 Mar 27;11(2):R42. doi: 10.1186/cc5730 (PMC2206463; doi:10.1186/cc5730)
Supplement: Additional file 2 — A Word document showing calculation of the ICU LOS using the postoperative prediction model. [file cc5730-S2.doc]

## Appendix B

Length Of Stay in the Intensive Care Unit (ICU) (LOS) with the postoperative prediction model can be calculated as:

LOS_postoperative_ (days) = 0.44 * 1.20^age/10 * 0.85^fev * 1.53^gerd * 1.32^vasc * 1.82^neu * 1.79^tte * 1.52^rec * 1.07^time * 1.14^col * 0.94^blood * 1.12^urin * 0.83^eda * 1.09^rmv * 1.03^peep * 1.58,

where age is patient's age per year; fev is the forced expiratory volume 1 (FEV1, see Table 1) in liters; gerd is 1, if patient has gastroesophageal reflux disease, 0 if not; vasc is 1, if patient has a vascular comorbidity, 0 if not; neu is 1, if patient has a neurological comorbidity, 0 if not; tte is 1, if transthoracic approach, 0 if transhiatal approach; rec is 1 if the patient had a colon reconstruction, 0 if stomach reconstruction; time is released operating time per minute; col is amount of colloids administration in liters; blood is amount of blood loss during the procedure in liters; urin is amount of urine produced during the procedure in liters; eda is 1, if Epidural Anesthesia (EDA) was administered, 0 if not; rmv was the maximum respiratory minute volume during the procedure in liters; peep is the highest positive end- expiratory pressure in cmH_2_O; 1.58 is the smearing factor.
