# Supplementary material for: Optimizing intensive care capacity using individual length-of-stay prediction models
Source: Crit Care. 2007 Mar 27;11(2):R42. doi: 10.1186/cc5730 (PMC2206463; doi:10.1186/cc5730)
Supplement: Additional file 3 — A Word document showing calculation of the ICU LOS using the intra-ICU prediction model. [file cc5730-S3.doc]

## Appendix C

Length Of Stay in the Intensive Care Unit (ICU) (LOS) with the intra-ICU prediction model can be calculated as:

LOS_intra-ICU_ (days) = 1.82 * 1.09^age/10 * 1.21^tte * 1.05^rmv * 1.31^chy * 1.83^ana * 1.71^oths * 1.97^pulm * 1.54^mi * 1.61^inf * 1.41^oth * 1.18,

where age is patient's age per year; tte is 1, if transthoracic approach, 0 if transhiatal approach; rmv was the maximum respiratory minute volume during the procedure in liters; chy is 1, if patient has a chylothorax, 0 if not; ana is 1, if patient has anastomosis leakage, 0 if not; oth is 1, if patient has an other surgical complication, 0 if not; pulm is 1, if patient has pulmonary complication, 0 if not; mi is 1, if patient has a myocardinfarction, 0 if not; inf is 1, if patient has an infection, 0 if not; oth is 1, if patient has an other non- surgical complication, 0 if not ; 1.18 is the smearing factor.
